# First *in vitro* evidence of modulated electro-hyperthermia treatment performance in combination with megavoltage radiation by clonogenic assay

**DOI:** 10.1038/s41598-018-34712-0

**Published:** 2018-11-09

**Authors:** Marjorie McDonald, Stéphanie Corde, Michael Lerch, Anatoly Rosenfeld, Michael Jackson, Moeava Tehei

**Affiliations:** 1 Centre for Medical Radiation Physics, University of Wollongong, Wollongong, NSW Australia; 2 Illawarra Health and Medical Research Institute, Wollongong, NSW Australia; 3 Radiation Oncology, Prince of Wales Hospital, Randwick, NSW Australia

## Abstract

Modulated electro-hyperthermia (mEHT) is a form of hyperthermia used in the treatment of cancer. It is a variation that relies on a particular form of enhanced selectivity to enable more effective cancerous cell death yet maintaining the integrity of healthy non-cancerous cells. It is yet to successfully make the major step into the wider medical community despite several encouraging trials. In this study, we investigate mEHT from an *in vitro* perspective. We demonstrate a supra-additive effect on 9 L gliosarcoma cells when exposed to mEHT in combination with MV X-ray radiation. The supra-additive effect is hypothesized to be induced by the mEHT mechanism that in turn causes apoptosis, membrane damage and an increase in rate of cell growth. This proves to be extremely advantageous in the case of the aggressive 9 L cell line as it is known to be radioresistant. However, the universal success of this multimodal treatment does not appear to be positive for all cell lines and requires further research. Due to the fundamental approach taken in this research, our results also provide a new prospect for mEHT to be a tool for sterilizing otherwise radioresistant cancers.

## Introduction

Hyperthermia uses active heating to treat cancer, either alone, or in combination with other modalities such as radiotherapy or chemotherapy^1,2^.

Despite conventional hyperthermia technology being used since the early 1900s, benefit is not widely accepted in conventional clinical practice. This is mainly attributed to hyperthermia’s limitation in transferring heat to deep tissue and its ability to focus this transfer of heat energy only on malignant cells^3^. Other limitations of hyperthermia include the limited correlation between temperature and the heat energy that is being delivered. This makes it problematic to control and supply sufficient energy to the target tissue.

Capacitive radiofrequency (RF) hyperthermia uses an alternative electric field to heat areas of the body and has been widely used in conventional practice in Korea and Japan. In Germany, it is most often used in complimentary clinics. A frequency of 13.56 MHz is often used as it provides reasonable penetration into the body without the need for electromagnetic shielding of the device and its public availability. mEHT differs from conventional capacitive heating in that a special fractal modulation of the carrier frequency is claimed to give enhanced the selection of the tumour cells in the target and allow the use of much lower applied power levels than other similar devices. It also may overcome the limited penetration of 13.56 MHz energy in human tissues especially through the subcutaneous fat and allow the treatment of sites such as the brain and close to the eye which can be difficult to treat with other external techniques. Tumour temperature is not measured directly but calculated form input power and other factors^4^. The actual penetration of the 13.56 MHz energy is disputed in the literature and the typical racial differences (until recent times) in subcutaneous fat distribution may partly explain the popularity of the different methods^5,6^.

The cell membrane maintains the integrity of cells by providing a barrier between the cell and the extracellular environment. mEHT has been designed to selectively autofocus electromagnetic power mainly on malignant cell membranes, which result in the breakage of the membranes^4^. The selection is based on the abnormality of the metabolic processes of cellular connections and the organising pattern of malignant cells from their corresponding healthy cells. Therefore, the characteristics mentioned above are believed to allow for the autofocus heating of the membrane rafts of malignant cells. In addition, the non-ionizing electromagnetic waves can penetrate into deep tissue, which allows for the maintenance of energy absorption in a desired locality^7^.

External beam radiotherapy is one of the predominant treatment modalities used today in cancer treatment. Higher megavoltage (MV) photon beams have been dominantly used in clinical oncology because of its effective treatment of deep-seated cancer, which makes MV beams the current interest among academic studies^8^. Radiation therapy uses high-energy electromagnetic radiation to shrink tumours and kill cancer cells^9^. Radiation therapy alone can destroy cancer cells by inducing significant damage (directly or indirectly) on their deoxyribonucleic acid (DNA). In many cases the damage to the DNA is short-lived as the DNA is naturally repaired. Extensive research has been invested into improving the efficiency in damaging the DNA.

The application of hyperthermia has been established as a complementary treatment to most of the traditional treatment modalities^10,11^. It has been shown to provide synergies with most of the traditional treatment modalities, which include radiation therapy^11–13^. Conventional hyperthermia combined with radiotherapy has been reported to improve clinical response, local control and the survival in randomised trials for patients with breast, head and neck cancers, skin melanoma and glioblastoma multiform^14,15^. However, it should be emphasized that there are many factors which can affect the overall complete response rate in patients^16,17^. The efficiency of radiotherapy is oxygen dependent. This means those tumours that are severely hypoxic are more resistant to radiation treatment. Therefore, one of hyperthermia’s role is to increase blood perfusion (oxygenation) of the tumour through the increased temperatures^18,19^. Hyperthermia is also more effective in hypoxic conditions. This subsequently increases sensitisation to radiation ionisation. The positive impact of hyperthermia in combination with radiotherapy is evident in clinical results^13,15,16^. However, there are disappointing clinical trials as well^20,21^. The controversial clinical results and unexplained underlying mechanisms including risks and safety issues of hyperthermia are its challenges in oncology. This multimodal application and its challenges are also extended to mEHT treatment. mEHT has been clinically shown to be successful as monotherapy as well as complementing with radiation therapy and chemotherapy^4^. Despite the positive trials on mEHT, medical communities soundly did not consider these results conclusive. Moreover, there are only a limited number of studies done *in-vitro*, thus it invites for more studies to be conducted.

In this study, we aimed to use clonogenic assay as a key experiment to help verify the effectiveness of mEHT treatment at the cellular level when combined with radiation. Aggressive gliosarcoma and metastatic breast cancer cell lines along with a non-cancerous cell line are utilised in this research. This will be the first study in mEHT to employ radiobiological clonogenic assay along with real time imaging to provide independent evidence on the efficiency of the treatment. The effectiveness of mEHT will be assessed both alone and in combination with radiation therapy.

## Results

### mEHT damages cell of 9L immediately after treatment but not on MCF-7 and MDCK

Confocal images with Propidium iodine (PI) staining were taken to test the selectivity of mEHT on the cancer cell. PI is a membrane impermeant dye used to detect dead cells or cells with compromised cell membranes.

**Figure 1:**
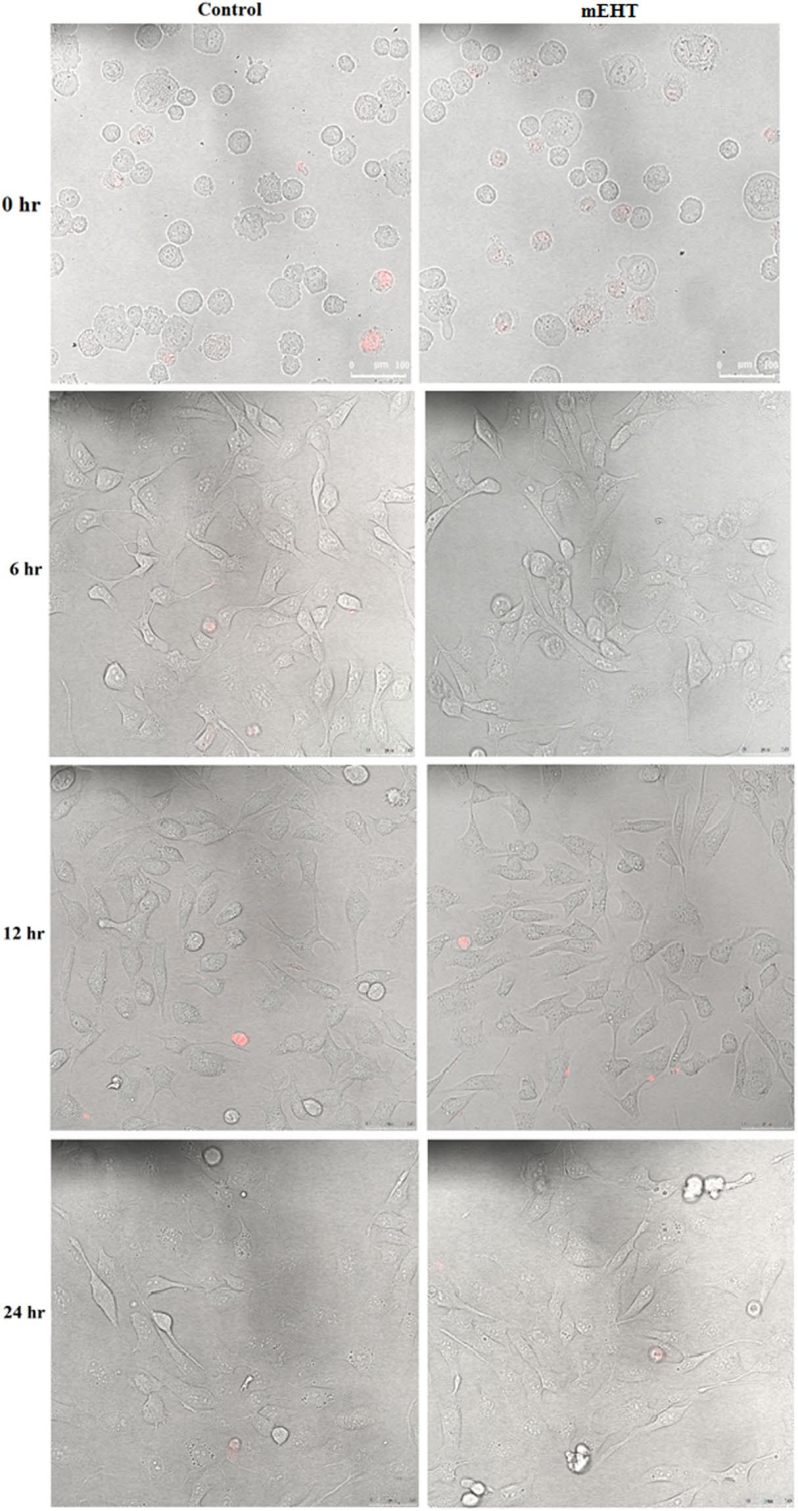
MDCK cells time-lapse images of cells with and without (control) 30-minute treatment of mEHT. Propidium iodine dye (fluorescing in red) was added to the cells immediately before taking the confocal microscope imaging. The assay was performed twice.

**Figure 2:**
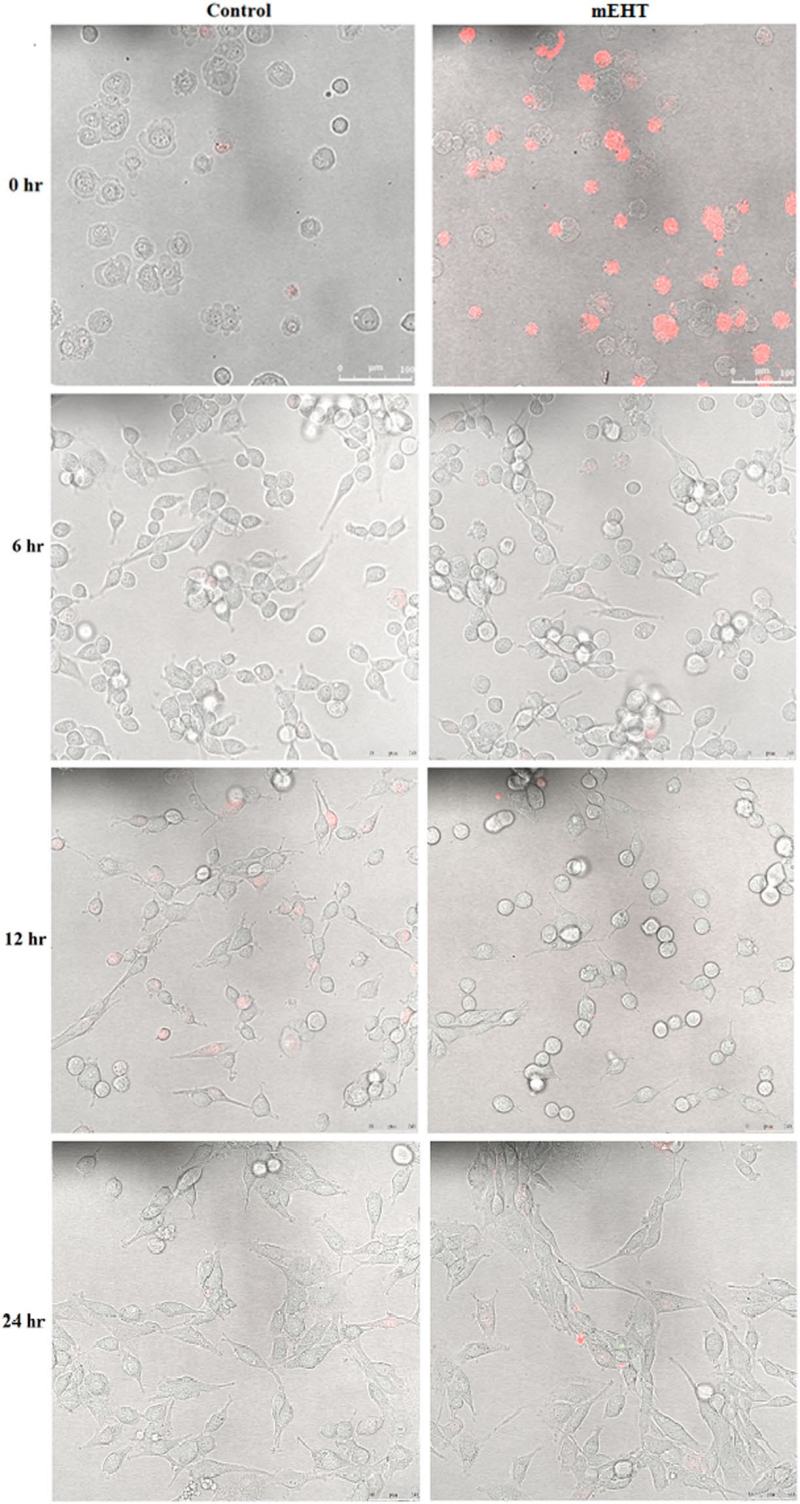
9 L cells time-lapse Images of cells with and without (control) 30-minute treatment of mEHT. Propidium iodine dye (fluorescing in red) was added to the cells immediately before taking the confocal microscope imaging. The assay was performed twice.

**Figure 3:**
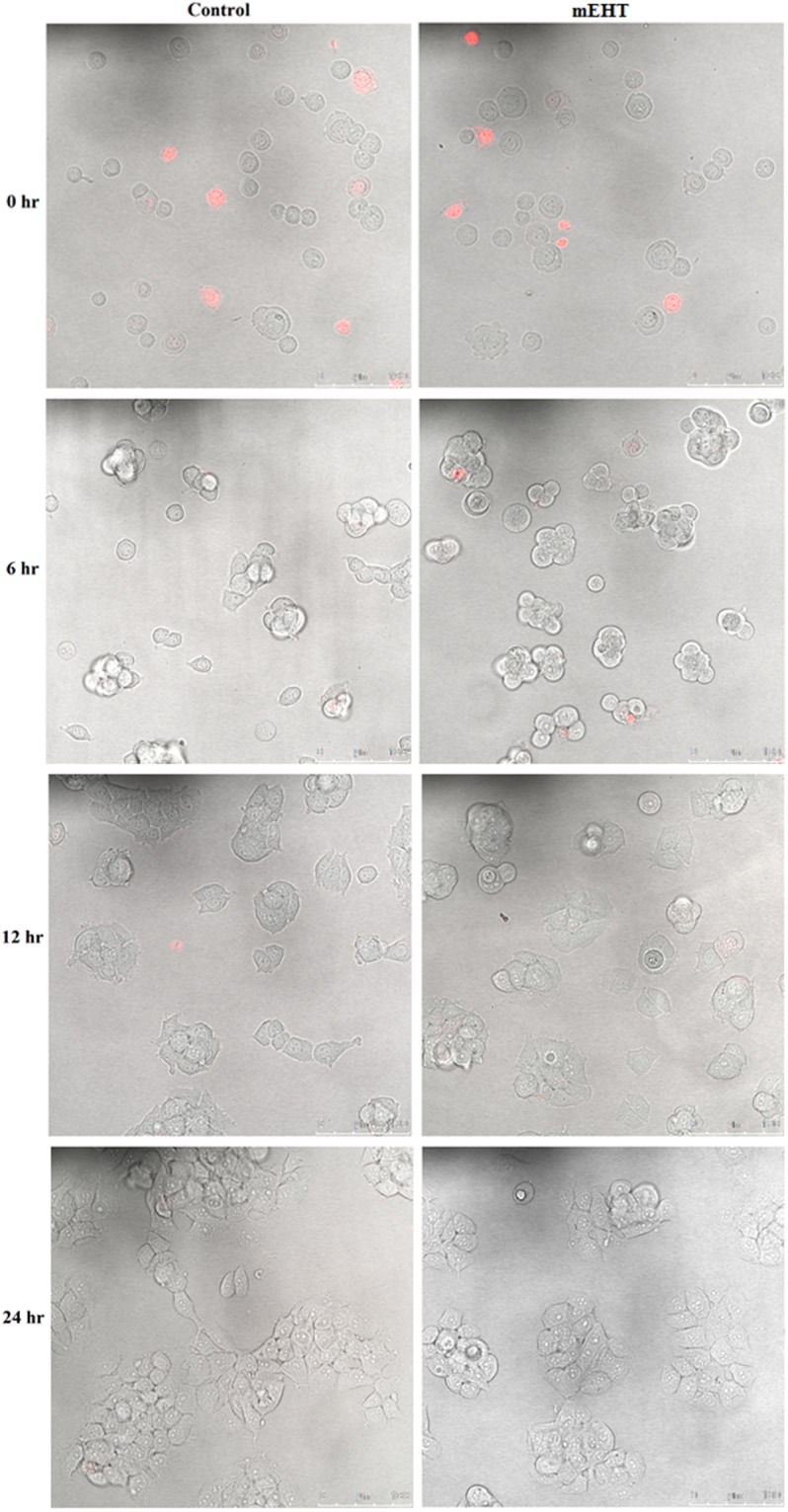
MCF-7 cells time-lapse Images of cells with and without (control) 30-minute treatment of mEHT. Propidium iodine dye (fluorescing in red) was added to the cells immediately before taking the confocal microscope imaging. The assay was performed twice.

The selective effect of mEHT is only observed in the 9 L cell line at the 0 hour time point (see Figs 1, 2 and 3). The 9 L cells appeared healthy in the images taken at 6, 12, and 24 hours. This implies that repair may take place shortly after the mEHT treatment. Conversely, damage induced by mEHT at the 0 hour was not observed for the MCF-7 results (see Fig. 3). As expected, there was no damage observed to the MDCK cell line (see Fig. 1) due to the selectivity of mEHT as reported in the literature for other non-cancerous cell lines^4^.

### mEHT induces change in cell growth and their growth rate

#### MDCK cell line

**Figure 4:**
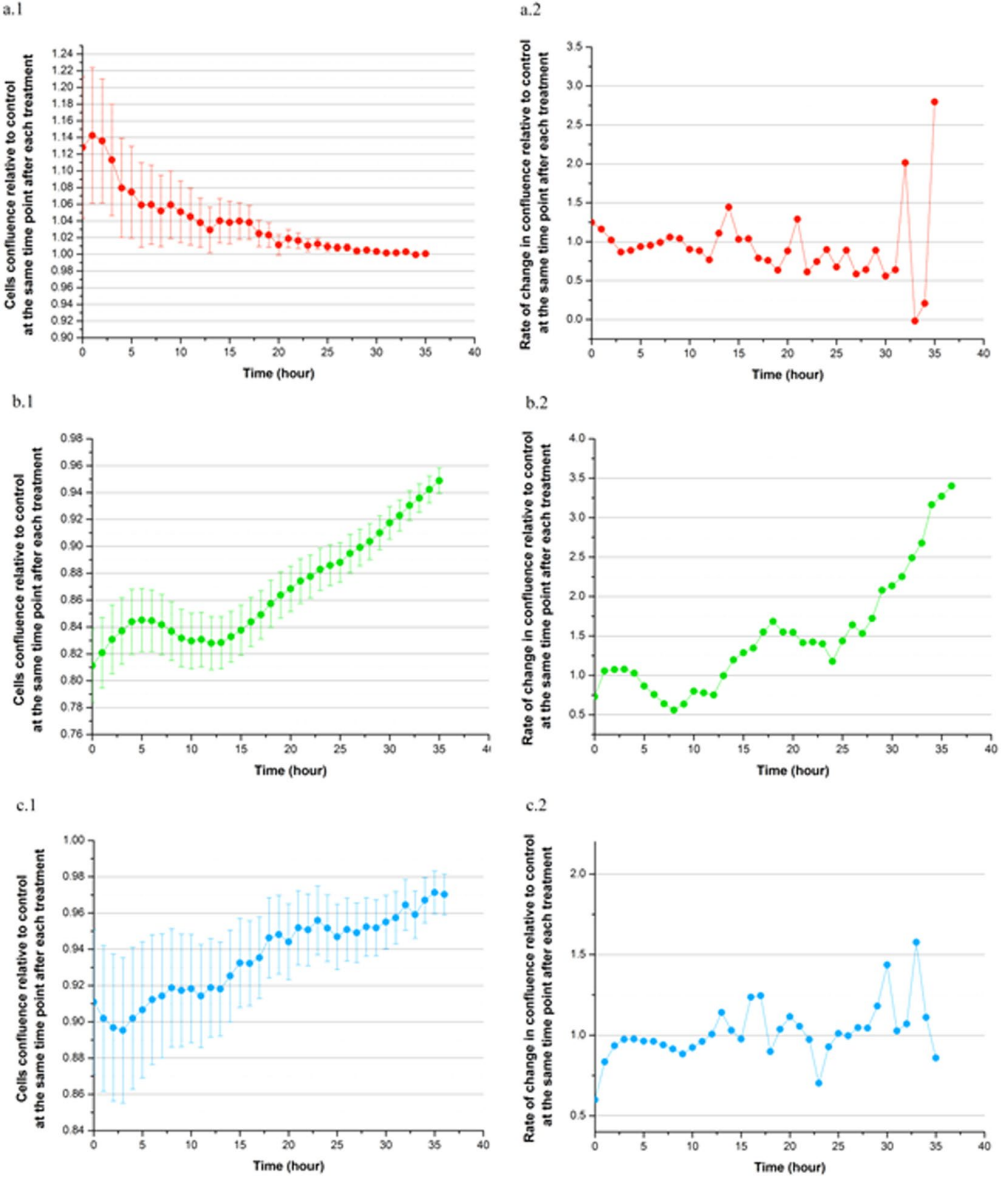
MDCK (**a**), 9 L (**b**) and MCF-7 (**c**) cell lines were plated in 36-well plates after mEHT treatment at the same starting density. After allowing cells to adhere to the plate for 2 hours, cells were then monitored for confluence in an IncuCyte imager over the timescale shown, where (a.1, b.1, c.1) treated cells confluence relative to control and (a.2, b.2, c.2) rate of change in treated cell confluence relative to control. The data was normalised to the control (untreated cells) for comparison of the curves. Results from six replicated wells are shown. Error bars indicate ± SEM. The 35-hour time period was chosen as it corresponds to the highest doubling time of our studied cells (i.e. 9 L).

After exposure to mEHT treatment, the trend for the curve of the cell growth of mEHT treated cells in the initial 12 hours is decreasing relative to the untreated cell control group (see Fig. 4a.1). It should be stressed that the actual confluence value is not decreasing for the mEHT treatment, but rather it is the value *normalised to the control group* that is decreasing. Within this 12-hour window, the response of the cell growth to the treatment is temporarily stagnant whilst the control group’s confluence increases in a stable manner. After 12 hours, the mEHT treated cells displayed an active response and this is evident in the more rapid fluctuation in the rate of cell growth (see Fig. 4a.2). Therefore, this results in the final growth curve converging to the same number of cells relative to the control group (shown in Fig. 4a.1).

#### 9L cell line

For this cell line, the mEHT treated cells’ growth curve is trending towards resumption of normal cell growth (see Fig. 4b.1). As the data was normalised to the control group, the results indicate the change in the cell proliferation (growth) rate. The rate of change in cell growth also increases in a near monotonic manner (see Fig. 4b.2). Hence this explains the phenomena of the observed cell growth curve as seen in Fig. 4b.1). Even if the proliferation is decreased just after the mEHT treatment, our results however indicate no added advantage for mEHT treatment alone on malignant 9 L cells in the first 35 hours post treatment.

#### MCF-7 cell lines

The data seems to be a combination of what was observed with the 9 L and MDCK cell lines. The mEHT treated group exhibited a similar trend for confluence outcome for the MCF-7 cell line. That is, long-term behaviour showed that the treated cells are converging to the control confluence value. This is evident in the trend of their cells growth curve (see Fig. 4c.1) and rate of change in the growth (see Fig. 4c.2). The convergence of the growth curve to the same number of control cells was detected at 35 hours, as shown in Fig. 4c.1. Nonetheless, the rate of change in the MCF-7 cells growth were relatively bounded compared to 9 L and MDCK (see Figs 4a.2 and 4b.2). This is seen by smaller fluctuations around the normalised mark (Fig. 4c.2) in comparison to 9 L (Fig. 4b.2) where the curve appeared to be monotonically increasing. This suggests that MCF-7 cells may have a higher resistance to mEHT treatment than 9 L cells over this time period.

As the mEHT treated cells provided a similar terminal confluence value (to the control group) in the 35-hour time period for both MCF-7 and 9 L, this confirms our findings that mEHT provides no increased cell death at the conclusion of this timeframe. However, the observed fluctuation in cell growth rate in the malignant cell line (9 L) induced by mEHT could be beneficial if the cells were more sensitive to radiation in the more rapidly growing phase.

### The selectivity of mEHT on malignant cells is not evident in the initial 96 hours

**Figure 5:**
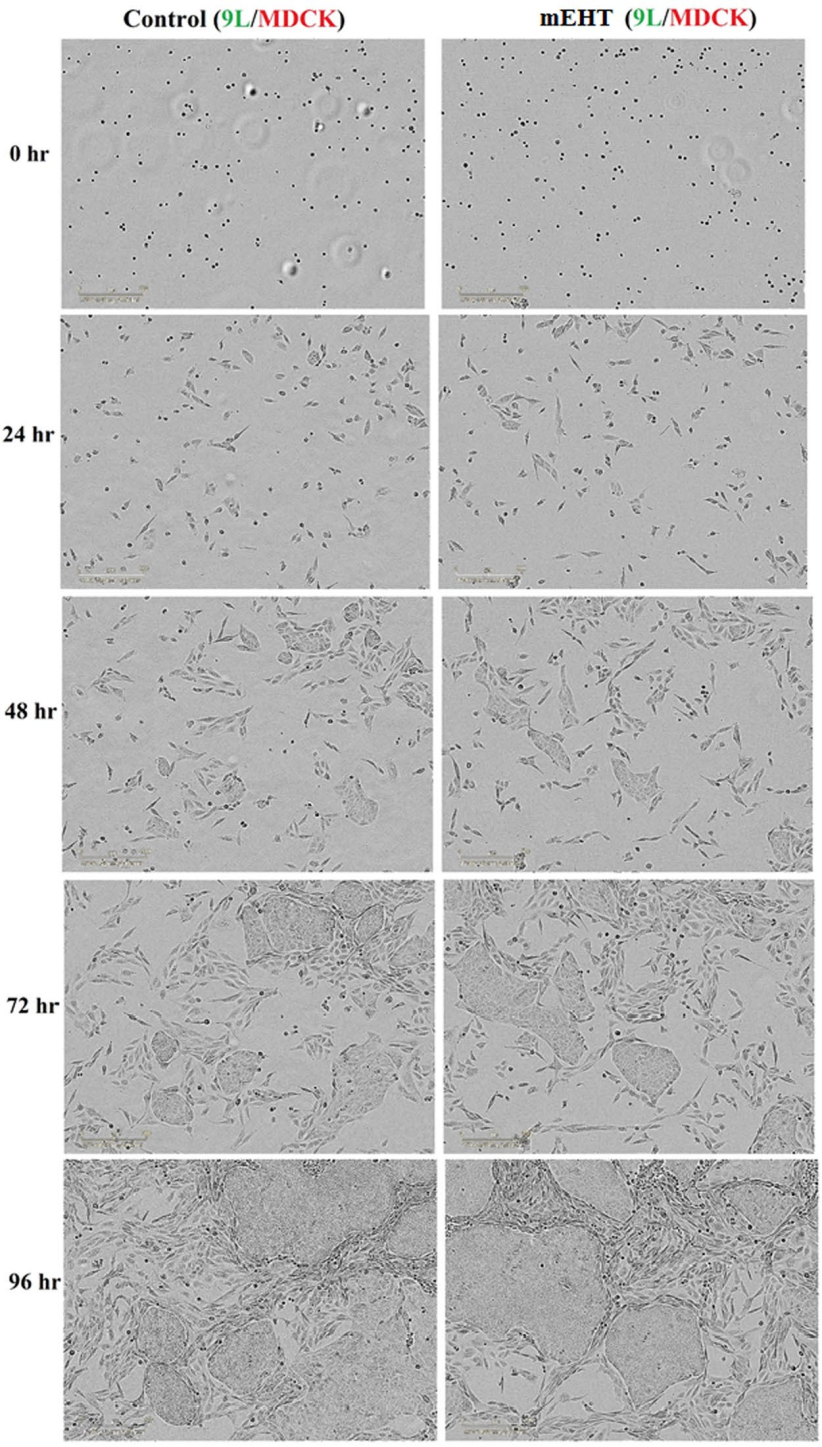
IncuCyte imaged of co-cultured of MDCK and 9 L cells with and without (control) mEHT treatment growth over a period of 96 hours. The same cells density was seeded into 24-well plates and images were taken every 24 hours. A representative example of the results from six replicated wells is shown.

**Figure 6:**
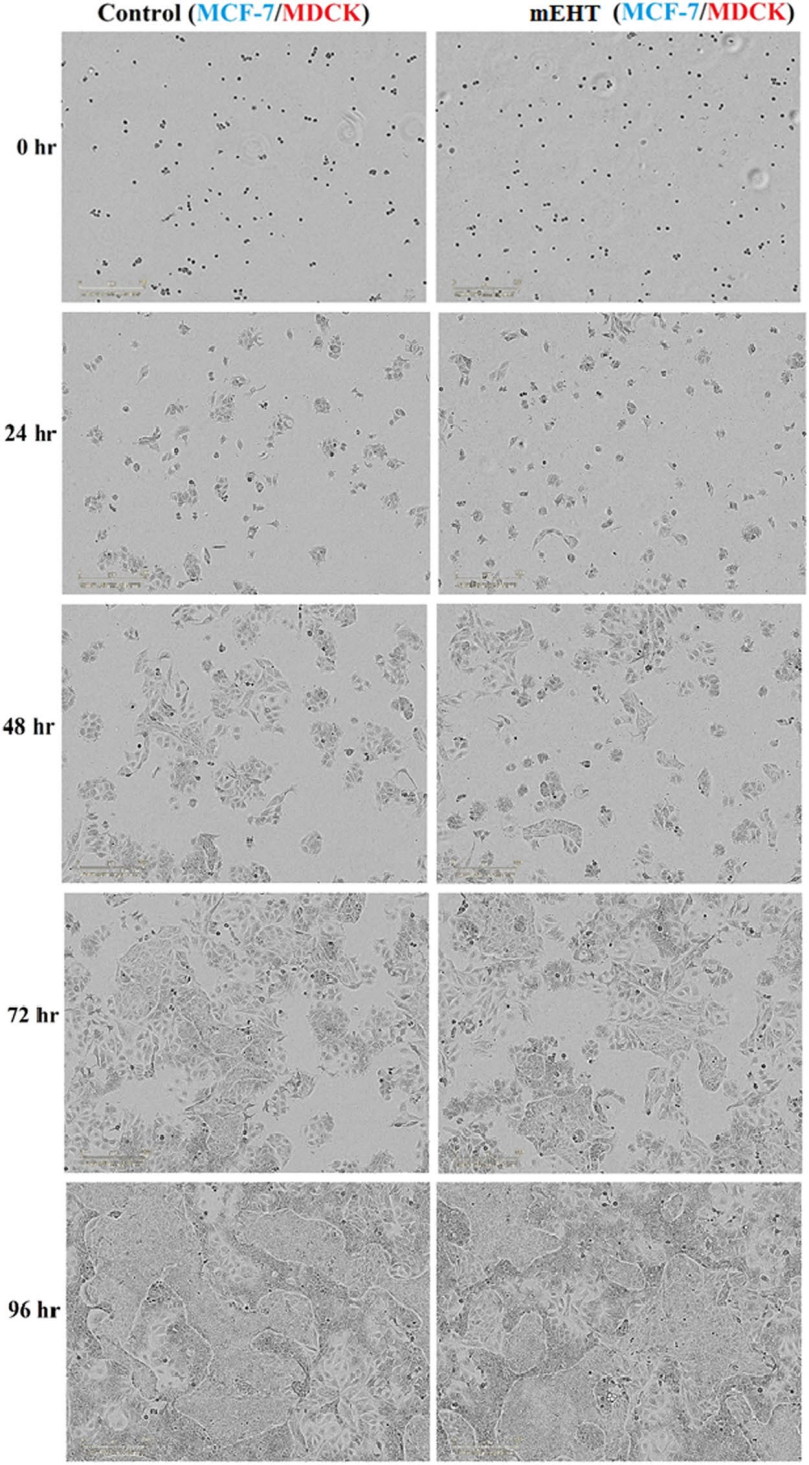
IncuCyte imaged of co-cultured of MDCK and MCF-7 cells with and without (control) mEHT treatment growth over a period of 96 hours. The same cells density was seeded into 24-well plates and images were taken every 24 hours. A representative example of the results from six replicated wells is shown.

This section incorporates real-time imaging to analyse beyond the time points of the results in 2.a) and 2.b). Additionally, we are co-culturing both the non-malignant and malignant cells to investigate the selectivity of mEHT in co-culture. As the co-cultured cells began to adhere to the surface and proliferate, their structure became more distinct which allowed for the distinguishing of the two cell lines in the IncuCyte images. This was first observed at the 48-hour mark (Figs 5 and 6). The 72-hour and 96-hour images displayed a co-existence of both MDCK and a particular malignant cell line (either MCF-7 or 9 L) in both the control and the mEHT treatment group, as shown in Fig. 5 (MDCK co-cultured with 9 L) and Fig. 6 (MDCK co-cultured with MCF-7). The co-existence demonstrated that the mEHT treatment did not have selectivity for the malignant cells within this extended timeframe.

### mEHT is not very effective as a sole method of treatment

**Figure 7:**
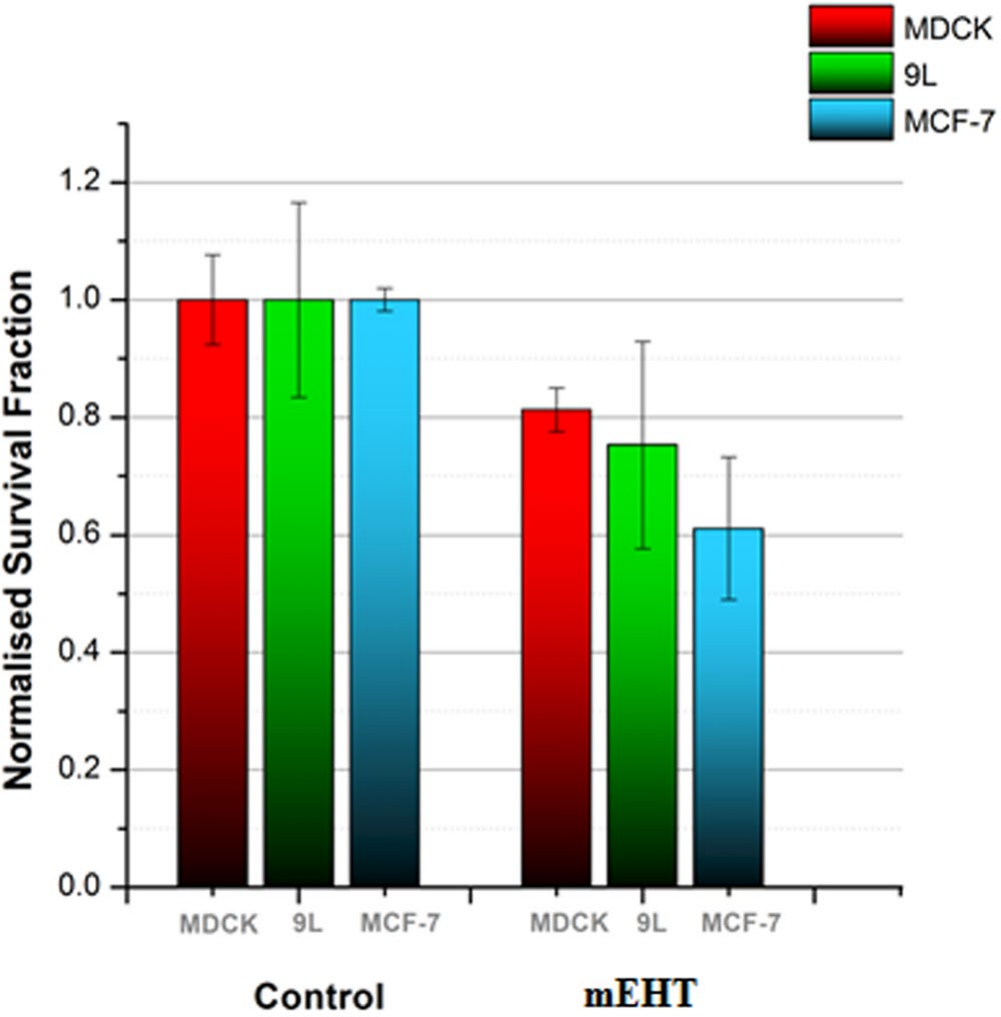
Normalised survival fractions of MDCK, 9 L and MCF-7 cell lines following 30-minute exposure to mEHT. Clonogenic assay was performed on both untreated (control) and treated cells for the three cell lines. The assay was performed at least 3 times. Errors bars represent the SEM.

The clonogenic assay results presented here, obtained after 15 doubling times of each cell line investigated show that the MDCK cells have a high survival fraction for the mEHT treatment at (81 ± 4)%. The 9 L cells showed a larger difference in the survival fraction compared to the control with a value of (75 ± 18)%. The MCF-7 cells showed a greater apparent sensitivity to the mEHT treatment, with a survival fraction of (61 ± 12)%. The difference in the survival fraction between the two malignant cell lines is within their respective uncertainties (refer to the error bars in Fig. 7). However, if the trend for reduced proliferation in MCF-7 compared to 9 L cells is real, this may due to mEHT inducing a short-term cell membrane damage for 9 L cells whereas it induced a more long-term impact on the cells’ biological properties (e.g., capacity to divide and proliferate) in MCF-7 cells.

### mEHT and radiation treatments have synergistic effects on 9L but not on MCF7 or MDCK

**Figure 8:**
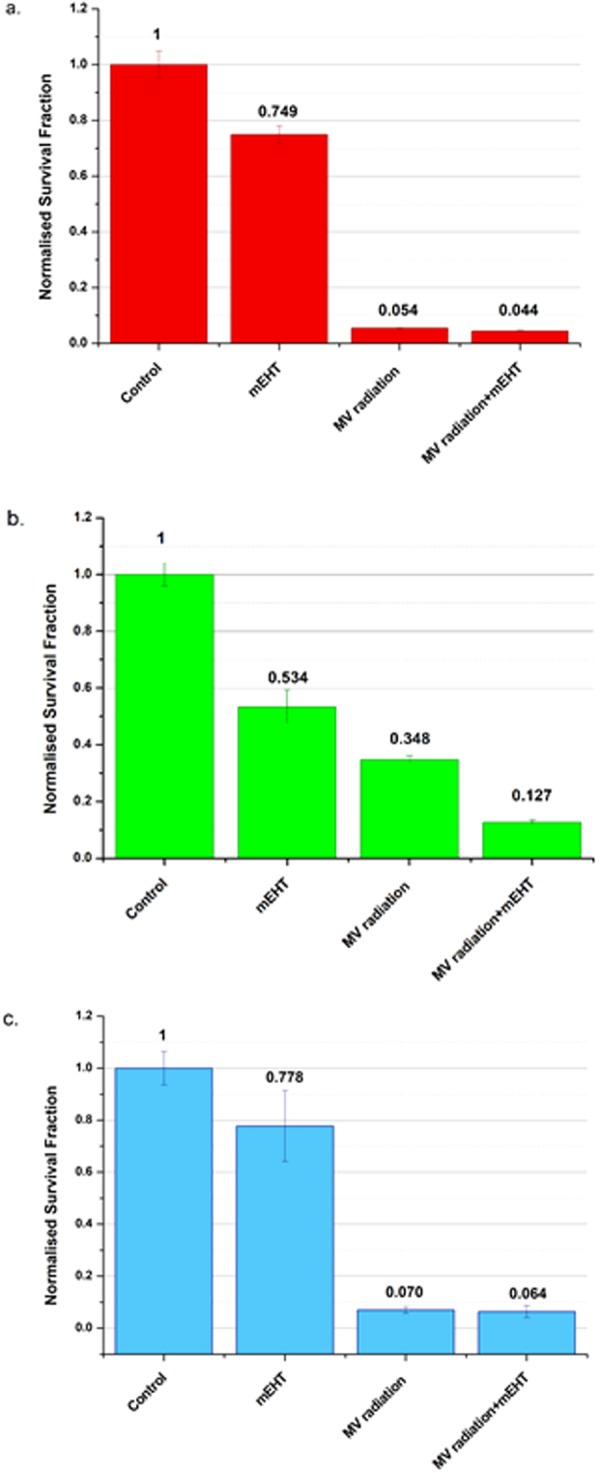
Clonogenic cell survival assay was performed on (**a**) MDCK (**b**) 9 L and (**c**) MCF-7 cells following 30-minutes exposure to mEHT and 10MV irradiation at 5 Gy, with and without individual components of the combination. The assay was performed twice. Errors bars represent the SEM.

The survival fraction results for MDCK showed a marginal contribution of mEHT pre-treatment when used in combination with a radiation dose of 5 Gy compared to the case where radiation is used alone (see Fig. 8a). The SF obtained for radiation alone and its combination with mEHT were (5.4 ± 0.2)% and (4.4 ± 0.2)% respectively. The SF of 9 L cells for radiation alone and its combination with mEHT were (35 ± 1)% and (13 ± 1)% respectively. This is a substantial decrease (Fig. 8b); indicating a supra-additive effect. The 9 L cell line is well known to be very resistant to radiation so this result makes it potentially clinically significant.

Despite the fact that MCF-7 showed a lower cell survival fraction than 9 L when treated with mEHT alone (see Fig. 7), a similar outcome is not observed in the mEHT combined with radiation treatment. This was expected because MCF-7 cells demonstrated resistance to mEHT as evident with the PI dye staining image (see Fig. 3) and the cells confluence rate of change (see Fig. 4c.2). The survival fractions of MCF-7 cells under exposure to radiation alone and mEHT treatment combined with radiation was (7 ± 1)% and (6 ± 2)% respectively (Fig. 8c). The results yielded no enhancement of the combined mEHT and radiation treatment on this radiosensitive malignant cell line.

## Discussion

mEHT kills malignant cells by apoptosis through external (membrane) signal path^22^. The images of mEHT treated cells with PI staining demonstrate the damaged membranes at the immediate time point (i.e., at the 0 hour) on the aggressive 9 L cells; however, no additional membrane damage was evident for the MCF-7 cell line when compared to the control without treatment. This suggests that the effect of mEHT treatment is not universal across different tumours cell lines. This is consistent with a reported set of results where it was shown that the mEHT treatment is effective only on the most aggressive cells lines^4^. However, this bias in which aggressive cell line it targets more effectively is not specified in the mEHT literature. Additionally, the time lapse images taken for both 9 L and MCF-7 after 0 hours indicate a different process that might be occurring within the cells as their membrane integrity was maintained right up to the 24-hour test period. This is inconsistent with the literature that demonstrated that mEHT destabilises the cell membrane and increases its permeability^4^. The observation in our results indicates a repair mechanism may have occurred shortly after treatment in our real-time image study (see Sections 2 (a) to (c)). This hypothesis was supported by the IncuCyte proliferating cell curves over a period of 35 hours after treatment (specifically, Section 2b)). For MCF-7, the fluctuation for the rate of growth change in the curve also indicates a potential repair process occurring (see Fig. 6b). The rate of change in the cells proliferation increases more rapidly towards the end of the 35-hour experiment period, which in this case corresponds to the confluence value converging to the control cell number in the mEHT treatment group as seen in Fig. 6a. The marginal deviations in the rate of change for cell growth seen in Fig. 6b for MCF-7 did not occur for 9 L, which exhibited a near monotonic increase in the rate of cell growth change (see Fig. 5b). Although this behaviour is vastly different to MCF-7, the ultimate outcome is the same: the cell confluence for both cell lines appear to be converging to the control value of 1, which in turn is the resumption of normal cell growth. Thus for a short-term timespan, there is no indication of an obvious advantage of mEHT as a standalone treatment method. These results contradict the findings for the mEHT cell damaging process, which has been repeatedly shown in numerous publications to date^4,23,24^. The referred damage process is the permanent damage in the cell morphology that in parallel caused the cells to undergo apoptosis within the initial 48 hours after the mEHT treatment^24^. At the time frame between 8 to 24 hours in the process is marked as the pivotal point where the cells are committed to die^24,25^.

According to the literature, the time delay in the selection and the tumour destruction of mEHT is due to apoptosis^23,26^. It has been repeatedly shown that the detection of apoptosis on the mEHT treated cells is evident^27,28^. Andocs *et al*. demonstrate this on U937 cell line using DNA fragmentation assay after 3 hours of treatment^27^. Yang *et al*. demonstrated this using FITC-conjugated Annexin V and propidium iodine reagents with different types of malignant cells 24 hours after treatment, which include MCF-7 and also a brain tumour cell line (U87MG)^28^. Our real-time imaging studies immediately post-treatment in Sections 2a) to 2c) are limited to assessing only the physical outcome. It is customised to observe the potential occurrence of apoptosis. However, clonogenic assay is our selected tool for assessing programmed cell death (i.e., beyond the possibility of cell reproduction). The results showed a lower survival fraction for both malignant (9 L and MCF-7) and healthy (MDCK) cell lines which indicate that cells underwent apoptosis. Although the clonogenic assay outcomes were positive in regards to inducing apoptosis for both malignant cell lines, it also unfavourably targeted the benign cell line, which raises some doubts on mEHT’s selectivity characteristic.

Experimental results reported by Szasz *et al*. on different cancerous cells co-cultured with non-cancerous cells demonstrated a complete disappearance of malignant cells by 24 hours in their most aggressive cell line, which is A431 squamous carcinoma cell line^4^. Our study did not show this same effect on the aggressive 9 L cell line that was co-cultured with MDCK cells for 96 hours as seen in Fig. 5. We hypothesise the occurrence of a stronger repair mechanism for 9 L compared to the A431 cell line in this time span. The A431 cell line is derived from epidermoid carcinoma in the human skin. Epidermoid carcinoma cells are generally radiosensitive and chemosensitive^29^. This suggests that the repair mechanism for the A431 cell line may not be as highly active in comparison to 9 L. MCF-7 cells are less aggressive than 9 L cells and thus the co-existence of MCF-7 with MDCK cells result in Fig. 6 is consistent with experimental results reported by Szasz *et al*. for their non- aggressive malignant cell line^4^.

Although the supposed benefits of mEHT as a sole method of treatment are moderately observed, the greater potential of mEHT lies in multimodal therapies (chemotherapy and hyperthermia is the most common multimodal treatment) context in treating malignant cells. The initial damage on the 9 L cells’ membrane (and potentially, the cell overall) as seen in the PI staining image in Fig. 2 immediately after mEHT treatment (i.e., at the 0 hour) creates a window of opportunity to integrate other treatment modalities. Thus, this marks the timing of our radiation to be near immediately (i.e., 15 minutes) after the mEHT treatment. This also marks the first instance of mEHT’s selectivity being observed in this research. In corresponding to the aforementioned observed initial damage of 9 L cell membrane at the 0 hour, the MDCK cell line is seen to be immune to the mEHT treatment. This is highlighted by no evident difference between the control group and mEHT treated group on MDCK (see Fig. 1).

For this research, the most significant sighting of selectivity from the mEHT treatment is observed when combined with 10 MV radiation in Section 2e). The observed selectivity is evident for the 9 L cells SF results. MCF-7 cells are radiosensitive but the effectiveness of the combined treatment was not expected in this study because the point of radiation (i.e., the timing) used was based on the results we obtained for 9 L and findings in literature^30^. In particular, the results from Section 2a) reflect this. It is possible that the absence of a lower survival fraction in MCF-7 in this multimodal treatment is due to the time gap between applying mEHT and radiation as data in section 2d) hints at a more long-term effect of mEHT.

The 9 L cells showed a supra-additive effect. We hypothesize that this is caused by mEHT allowing for the initial damage to the cancerous cells, which in turn allow for the free radicals generated from radiation to freely enter the cells and effectively induce cell death (i.e., apoptosis). This is a significant result because 9 L is a type of tumour that is closely related to glioblastoma, the most fatal form of brain cancer^31^. Additionally, there is currently no effective treatment for this tumour type, not even in multimodal treatments. The experimental results find mEHT to complement radiotherapy quite well. This is consistent with the literature that shows that mEHT therapy is most effective when combined with conventional treatments^4,26^. Hence, this proves to be a promising candidate of treatment for this type of cancer (i.e., glioblastoma).

Moreover, our results demonstrate a new exciting prospect for mEHT treatment. In particular, mEHT induced a rapid increase in the cell growth rate of the radioresistant 9 L cell line. This is significant because mEHT appears to provide a new tool to manipulate the cells’ growth rate. This in turn modifies these types of radioresistant cells to be more radiosensitive, which allow them to be a stronger candidate for radiotherapy. Recall that one of fundamental laws of radiobiology states that an increased cell growth rate will result in greater radiosensitivity in the tissue. Thus, we believe that this new tentative path for mEHT research should be pursued more actively.

All the mEHT literature results demonstrate only a short-term response in the cells to the treatment *in vitro*. The experiments and analyses conducted in this work focused on the physical outcome of the mEHT treatment on the cells. The main component of this research is to assess the effectiveness of the mEHT treatment based on the cells’ radiobiological “endpoint”, which has never been performed before. The two main experimental methodologies employed in this research are real-time imaging and clonogenic assay. The aforementioned methodologies are less dependent on artefacts, as opposed to the methods utilised by other mEHT studies. The advantage of this is that the results we obtain are interpreted more easily. In particular, with clonogenic assay as this is considered the gold standard in radiobiology.

In summary, no benefit of mEHT treatment alone was observed in this study. However, the combined mEHT treatment with radiation therapy on the most aggressive and radioresistant malignant cells produced a significant increase in cell death. The results show the potential of this combined treatment modality in some, but not all, tumours cell lines. The most significant insight gained from the mEHT treatment is in its apparent potential to manipulate the cell growth rate. Specifically, on the radioresistant 9 L cell line (which is closely related to glioblastoma). This new aspect of mEHT treatment shows promise as the cells’ growth rate is closely tied to its radiosensitivity. Further study to support the results ascertained is encouraged to fully establish whether mEHT has a direct influence on the cell growth rate. Additionally, the present work provides the first set of independent data that is not based on external results or methods from other literature in the field. Future research also could include the investigation and analysis of this combined treatment method and the use of other cell lines.

## Materials and Methods

### Cell Culture

Gs-9L rat gliosarcoma cells originated from an N-nitrosomethylurea-induced tumour and were established by the European Collection of Cell Cultures. Madin-Darby canine kidney is a non-cancerous cells line (MDCK), derived in 1958 by S.H. Madin and N.B. Darby from the kidney tissue of an adult female cocker spaniel. MCF-7 cells line is derived from a pleural effusion from a patient with metastatic breast cancer. Cell cultures were maintain in Dulbecco’s Modified Eagle medium (DMEM, Gibco BRL, AUS) containing 10% (v/v) fetal bovine serum (FBS, Sigma-Aldrich, MO, USA) and 1% (v/v) penicillin/streptomycin (Pen Strep, Gibco BRL, AUS) in a T75 cm^2^ BD Falcon^TM^ Tissue Culture Flasks (Franklin Lakes, NJ, USA) and incubated at 37 °C in a humidified and 5% (v/v) CO_2_ atmosphere.

### mEHT treatment procedure

Cells were grown and passaged using T75 cm^2^ BD Falcon^TM^ Tissue Culture Flasks. Confluent cells were washed with Phosphate-Buffered Saline *(*Ca^2+^/Mg^2+^ free) (PBS, Gibco *BRL*, *AUS)*, detached using Trypsin- ethylenediaminetetraactic acid (Trypsin-EDTA, Gibco BRL, AUS) and rediluted in complete DMEM before evenly distributed into 2 ml cryogenic tubes. The mEHT sample tubes were exposed to mEHT via the LAB-EHY 100 laboratory unit and *in vitro* applicator (Oncotherm GmbH, Germany) at 42 °C for 30 minutes. Temperature was controlled real time via calibrated thermocouples. The control sample tubes were at room temperature during the short time between getting the samples from one incubator to the next. This practice is carried out to maintain a controlled environment from the time the treatment samples leave the incubator (set at 37 °C) and returning to the incubator (also set to 37 °C) after the treatment has been applied. Apart from very short transit period, all the samples (both control and treatment) are cultured in the incubator at the definite 37 °C.

### Confocal Microscope Imaging of cells stained with Propidium iodine

The cells were seeded and grown in a ^TM^Lab-Tek^TM^II Chamber Slide^TM^System 4 wells (growth area = 1.8 cm^2^) after applied mEHT treatment. The cells were incubated at 37 °C until taken for imaging using a Leica confocal laser scanning microscope. Immediately prior to imaging, the cells were washed with PBS (Ca^2+^/Mg^2+^ free), and 200 mL PI master mix (100 mg/ml RNase A, 40 mg/mL PI, and PBS pH 7.4) was added to the cells. Light and fluorescence microscope images were obtained using a Leica confocal laser scanning microscope (Leica TCS SP5 Advanced System – UV-VIS-IR and X1-Port Access with SMD FCS and CO_2_ incubation chamber, Germany) with oil immersion objective lens and at the excitation of 488 nm.

### IncuCyte Imager and data analysis

IncuCyte images were taken to assess the growth rate (proliferation) of the cells pre-treated with mEHT. The results were normalised to the control (untreated cells). This experiment was carried out to investigate if the apoptosis induced by mEHT affects the cell proliferation during the period shortly after the treatment. Each of the recorded data values are normalised to their corresponding control value to give a better comparison between the treatment group results. The errors bars are standard errors of the means (SEM). The experiments were performed three times independently.

### Co-culture

The key claim in mEHT treatment is the selectivity function on malignant cells. IncuCyte imager was employed to assist in this assessment. Prior to the mEHT treatment, the malignant (9 L or MCF-7) and non-malignant (MDCK) cell lines are transferred into the same tube. The type of tube and the volume of the cell solution is the same as used in all other experiments previously described. Similarly, the mEHT treatment setup is the same as the other experiments. After mEHT treatment, the co-cultured non-malignant cells (MDCK) and malignant cells (9 L or MCF-7) at the same cell density as the controls (co-cultured cells without mEHT treatment) were seeded into the wells and imaged every 24 hours over 96 hours. Different cell lines have different proliferation rates (doubling time) and this was accounted for in the preparation of the co-cultured cells prior to apply the mEHT treatment for imaging. Results are as shown in Fig. 5 for co-culture of MDCK and 9 L cells and in Fig. 6 for co-culture of MDCK and MCF-7 cell.

### Irradiation procedure and set up

mEHT pre-treated cells and untreated cells were irradiated with 10 MV photon beams from a Versa HD linear accelerator (Elekta, Stockholm, Sweden) at a dose of 5 Gy. We selected a gap of 15 minutes between the mEHT treatment and the exposure of radiation taking into account two factors. The first is based on the PI staining results of 9 L. The second is we deemed 15 minutes to be a feasible time gap when considering a clinical treatment. The cryogenic tubes were placed vertically at a depth of 2.2 cm in solid water to match the maximum depth dose of the 10MV photon field. To maintain full scatter conditions, 10 cm of solid water was placed behind the flask with 2 cm solid water around its sides.

### Clonogenic Assay Survival Fraction and data analysis

The efficiency of the treatments was assessed using clonogenic survival assay as the radiobiological endpoint. The clonogenic cell survival assays is employed to determine cell death through loss of reproductive integrity and the ability to proliferate indefinitely.

Cells were plated after the applied treatment at low densities in 100 mm tissue culture dishes containing 10 ml of complete DMEM. Each sample involved a minimum of three cells densities with triplicate dishes for each density. After 15 doubling times, the colonies in the dishes were fixed and stained with a solution of 25% crystal violet and 75% ethanol.

A colony was considered as surviving if it contained more than 50 cells. The plating efficiency (PE) was calculated as the number of surviving colonies divided by the number of cells seeded. The number of colonies that arise after treatment, which is expressed in terms of PE, is called the cell clonogenic survival fraction (SF):

$$SF\,(treated\,cells)=\frac{PE\,(treated\,cells)}{PE\,(untreated\,cells)}$$

The errors bars are standard errors of the means (SEM). The assays of all treatment conditions (with or without mEHT/radiation treatment) were performed a minimum of three times independently.

## Data Availability

No specific data availability circumstances are required for this work. All experimental protocols are self-contained, and the relevant datasets are disclosed throughout the article.
